# Gene Expression Profile of Benign, Intermediate, and Malignant Spitz and Spitzoid Melanocytic Lesions

**DOI:** 10.3390/cancers16101798

**Published:** 2024-05-08

**Authors:** Alessio Giubellino, Yuyu He, Sarah A. Munro, Yan Zhou, Kyu Young Song, Jose A. Plaza, Carlos A. Torres-Cabala, Andrew C. Nelson

**Affiliations:** 1 Department of Laboratory Medicine and Pathology, University of Minnesota, Minneapolis, MN 55455, USA; hexxx876@umn.edu (Y.H.); zhou1237@umn.edu (Y.Z.); songx047@umn.edu (K.Y.S.); nels2055@umn.edu (A.C.N.); 2 Masonic Cancer Center, University of Minnesota, Minneapolis, MN 55455, USA; 3 Minnesota Supercomputing Institute, Minneapolis, MN 55455, USA; smunro@umn.edu; 4 Department of Pathology, The Ohio State University Wexner Medical Center (OOSUWMC), Columbus, OH 43210, USA; josea.plaza@osumc.edu; 5 The University of Texas MD Anderson Cancer Center, Houston, TX 77030, USA; ctcabala@mdanderson.org

**Keywords:** Spitz, Spitzoid, RNA-sequencing, nevus, melanoma

## Abstract

**Simple Summary:**

The interpretation of Spitz and Spitzoid melanocytic lesions can be challenging, even among experts in the field. The discovery of genomic aberrations has contributed to better defining these lesions but knowledge gaps remain. Here, we present gene expression analysis of RNA sequencing data as an additional molecular tool to contribute to filling these gaps and better classifying these lesions. By gene expression profiling, we were able to identify distinct categories, suggesting that the use of this tool may help to improve the characterization of these lesions.

**Abstract:**

Spitz and Spitzoid lesions represent one of the most challenging melanocytic neoplasms in dermatopathology. Nosologic classification has been more recently improved by the discovery of novel molecular drivers, particularly translocations. In the current study, we aimed to use an unbiased approach to explore the gene expression profile of a group of melanocytic Spitz and Spitzoid melanocytic lesions ranging from benign lesions to melanoma, including intermediate lesions such as SPARK nevi and atypical Spitz tumors/melanocytomas. Using unsupervised analysis of gene expression data, we found some distinct hierarchical clusters of lesions, including groups characterized by *ALK* and *NTRK* translocations. Few non-ALK translocated tumors demonstrated increased ALK expression, confirmed by immunohistochemistry. Spitz tumors with overlapping features of dysplastic nevi, so-called SPARK nevi, appear to have a common gene expression profile by hierarchical clustering. Finally, weighted gene correlation network analysis identified gene modules variably regulated in subtypes of these cases. Thus, gene expression profiling of Spitz and Spitzoid lesions represents a viable instrument for the characterization of these lesions.

## 1. Introduction

Melanocytic tumors with Spitzoid morphology represent a challenging share of melanocytic pathology and their nosological interpretation is frequently problematic even among experienced pathologists specializing in the interpretation of these lesions [1].

Recently, molecular studies of these neoplasms have shed light on underlying mechanisms of tumorigenesis and have allowed not only better classification but also provided important prognostic information [2].

Within this category of melanocytic lesions, there is a spectrum ranging from benign Spitz nevi to Spitzoid and Spitz melanoma, passing through intermediate lesions identified as “atypical Spitz tumors” or melanocytomas [3]. There are also related lesions that may represent variations in these three categories. For example, pigmented spindle cell nevus of Reed represents a variation in benign Spitz nevi with heavy pigmentation and we now understand that many of these lesions carry an NTRK translocation as the distinctive molecular feature characterizing them [4]. Similarly, Spitz nevi can present with dysplastic histology features and these lesions are sometimes identified with the term “melanocytic nevi with features of Spitz nevi and Clark’s/dysplastic nevi” (or “SPARK nevi”) [5]. Other lesions that were originally included in the Spitz family, such as BAP1-inactivate nevi, are now classified separately [6].

A landmark study of Spitz neoplasms [7] about a decade ago found that around half of Spitz lesions carry a fusion and that these molecular characteristics are generally mutually exclusive. The most common genes involved in these fusions include ALK, ROS1, and NTRK with different translocation partners [1,2]. Less common genes involved in these translocations include BRAF, MET, and RET [8], among others. Moreover, we now understand that the presence of common gene mutations usually found in regular nevi and melanoma, such as BRAF mutations, exclude a diagnosis of Spitz lesion [9]. These studies have also informed the new WHO classification of these lesions, specifically the removal from the category of Spitz of melanocytic lesions that carry BRAF or NRAS mutations [10,11].

Several different techniques are used to help in classifying challenging melanocytic lesions, like Spitz tumors, into a benign, intermediate (melanocytoma), or malignant category. These techniques include fluorescence in situ hybridization (FISH), comparative genomic hybridization (CGH), and next-generation sequencing (NGS). The combination of molecular information from such techniques and a consensus among pathologists usually allows us to classify these lesions at best, although sometimes, the results are variable and inconclusive (e.g., borderline FISH results) [12,13].

The use of next-generation DNA sequencing and gene expression analysis has improved the classification of different types of melanocytic lesions and improved outcomes in this patient population [14]. This further emphasizes the limitations of using morphology alone for the correct interpretation of these lesions.

We performed targeted transcriptome RNA sequencing of 1385 cancer-related genes in a spectrum of melanocytic lesions ranging from benign Spitz and Spitzoid nevi to Spitzoid melanoma, with some intermediate lesions (“Spark” nevi, atypical Spitz or Spitzoid tumors/melanocytomas) and we present our results in the present study.

## 2. Materials and Methods

Specimen selection: A total of 25 Spitz and Spitzoid neoplasms were identified from the pathology archive of the University of Minnesota in a search ranging from January 2015 to June 2019. IRB approval was obtained. Clinical data were retrieved using our electronic medical record (Epic Systems, Verona, WI, USA).

These lesions were evaluated by three board-certified dermatopathologists (A.G, C.T.B, and J.P.) with a consensus morphology-based diagnosis and categories were segregated based on morphologic assessment prior to analysis. Histopathologic criteria for each category of melanocytic lesions are detailed in the last WHO Classification of Skin Tumours, Fourth Edition [11].

RNA-sequencing: Unstained slides were macro-dissected to collect the tumor. Total RNA was extracted and purified using an RNeasy FFPE Kit (Qiagen, Germantown, MD, USA). RNA samples were then quantified and analyzed for quality (Agilent RNA 6000 Nano Kit). Library preparation and targeted gene enrichment were performed with the TruSight RNA Pan-Cancer Panel Kit according to the manufacturer’s protocol (Illumina, San Diego, CA, USA). Libraries were sequenced on the Illumina NextSeq 550 System. FASTQ file analysis was performed using the Illumina BaseSpace RNA-Seq Alignment Application 2.0.2. This is a pipeline developed and supported by Illumina. The pipeline uses Bowtie 0.12.9 to filter unwanted sequences such as rRNA and mitochondrial RNA [15]. Then, STAR 2.6.1a is used to map the filtered sequences against the reference genome [16]. Manta 1.4.0 is used for fusion calling and Strelka v. 2.9.9 is used to call SNVs [17,18]. In the last step of the pipeline, Salmon 0.11.2 is used to quantify and merge transcripts to create gene-level counts [19].

Annovar [20] (2018-04-15 version) was used to annotate the variant call files with clinical genomic information including gnomAD minor allele fractions, COSMIC cancer listings, and NCBI ClinVar clinical significance. Annotated VCFs were then used with D3Oncoprint. Quality control of variants was performed based on the distribution of allele depth. We kept the variants that meet the following criteria: (1) alternate allele depth (AD) > 5 and VAF ≥ 5% or (2) AD = 4 or 5 and VAF ≥ 15%. We also excluded small indels in repetitive sequences with VAF < 10%. Annotated and quality-filtered variant calls were reviewed by a board-certified molecular pathologist (ACN) for potential or known clinical significance using established professional guidelines [21]. The resulting filtered and prioritized VCFs were analyzed with D3Oncoprint to create mutation heatmaps. We also used the ToppGene Suite [22] for functional enrichment analysis.

Gene expression analysis: Gene-level counts from the BaseSpace RNA-Seq Alignment Application were analyzed using custom R scripts and open-source R packages. In a typical whole transcriptome gene expression analysis study, we would remove low expressed genes but because this data set was limited in scope due to the size of the targeted capture, we used count data from all 1385 genes in the Illumina Trusight Pan Cancer Panel. For differential gene expression analysis, count data were analyzed using the edgeR package (CITE edgeR) normalized using the “RLE” method from edgeR. Differential expression testing was performed for each comparison with the glmQLFit and glmQLFTest functions from edgeR. Genes were reported as significantly differentially expressed if the FDR value was <0.05. For Weighted Gene Co-Expression Network Analysis (WGCNA) [23], we used the R package WGCNA [24]. Count data were normalized using voom. Sample hierarchical clustering was performed with the hclust function using method = “average”. We used clusterProfiler [25] for GO term enrichment over-representation analysis of the gene modules [26]. A q-value threshold of 0.10 was applied; this is a more relaxed threshold than the typical 0.05 threshold to allow for more possible GO terms to be provided for gene module identification purposes. Module gene lists were uploaded to the String-Db protein–protein interaction network analysis tool for additional functional annotation and analysis [27].

NTRK and ALK Immunohistochemistry (IHC): Skin biopsies were fixed in formalin and embedded in paraffin. Four-micron-thick sections of the tissues were cut and mounted on frost-free glass slides. The tissue sections were processed for IHC using BenchMark Ultra analyzer (Roche diagnostics, Indianapolis). The prediluted antibodies used were pan-NTRK (clone EPR17341) and ALK (clone D5F3) manufactured by Roche Diagnostics. Briefly, after blocking the endogenous peroxidases, the antigen retrieval was performed using CC1 (Roche diagnostics) followed by antibody incubation for 32 min for pan-TRK. ALK immunohistochemistry was performed as directed by the vendor. The antibody signal was detected using an Opti View detection kit and counter-stained with hematoxylin and cover slipped.

## 3. Results

**Figure 1:**
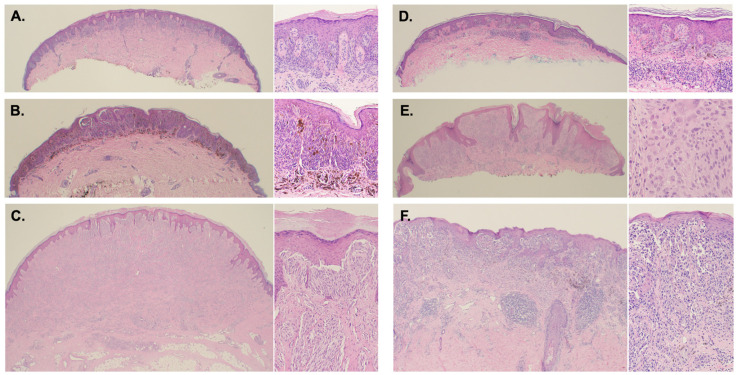
Representative histopathology pictures of selected cases from each category. (**A**) Compound Spitz nevus; (**B**) Reed nevus with NTRK-MYO5A translocation; (**C**) ALK-fusion Spitz nevus (TMP3-ALK); (**D**) “Spark” nevus; (**E**) Atypical Spitz tumor; (**F**) Spitzoid melanoma. Low power images magnification is at 4× and high power images are at 20× magnification.

Patient characteristics and histopathology. The melanocytic lesions in our cohort (total number = 25) were classified, following a consensus, into one of the following categories: (1) Spitz nevus (SN), including compound Spitz nevus and desmoplastic Spitz nevus (*n* = 5); (2) pigmented spindle cell nevus of Reed (RN) (*n* = 2); (3) Spark nevus (SPARK) (*n* = 5); (4) atypical Spitz tumor (AST) (*n* = 5); and (5) ALK translocated Spitz nevi (ALK) (*n* = 2); (6) invasive melanoma (MM) with spizoid features (*n* = 3); we also identified three “compound Spitz nevi with atypia” (SNa) that could not be classified otherwise. Table 1 illustrates the demographic, histologic, and follow-up data for all the lesions used in this study. Figure 1 shows the histopathologic images of representative cases from each category.

Translocations and mutation analysis. We performed RNA sequencing using RNA extracted from all the 25 lesions in our cohort. The average percent of targeted genes with >30× coverage was 58.31% (standard deviation of 6.41%) and the average percent of aligned reads was 97.45% (standard deviation of 0.59%).

Two compound Spitz nevi (ALK1 and ALK2) demonstrated ALK fusions (TPM3-ALK rearrangement for both lesions) (Table 1) and showed characteristic histomorphology for this category of tumor, i.e., fascicles of spindled melanocytes with plexiform growth pattern, in addition to a dome-shape clinical appearance (Figure 1). Two other lesions diagnosed on morphology as pigmented spindle cell nevi of Reed (RN1 and RN2) demonstrated a fusion in the NTRK3 gene (MYO5A-NTRK3 rearrangement for both lesions), a known molecular finding present in 50–60% of these lesions.

In addition, one of the melanomas (MM1) was found to have a V600K mutation in the BRAF gene; this molecular finding specifically excludes the diagnosis of Spitz melanoma and, given the morphology, is best classified as Spitzoid melanoma.

Sequence mutation analysis identified a large amount of mutation variability, although the majority of these mutations were classified as “mutations of undetermined significance”. Among the true pathogenic mutations, we found a mutation in HRAS (Q61K) in a desmoplastic Spitz nevus and in an atypical Spitz tumor, confirming the Spitz lineage of these lesions.

**Figure 2:**
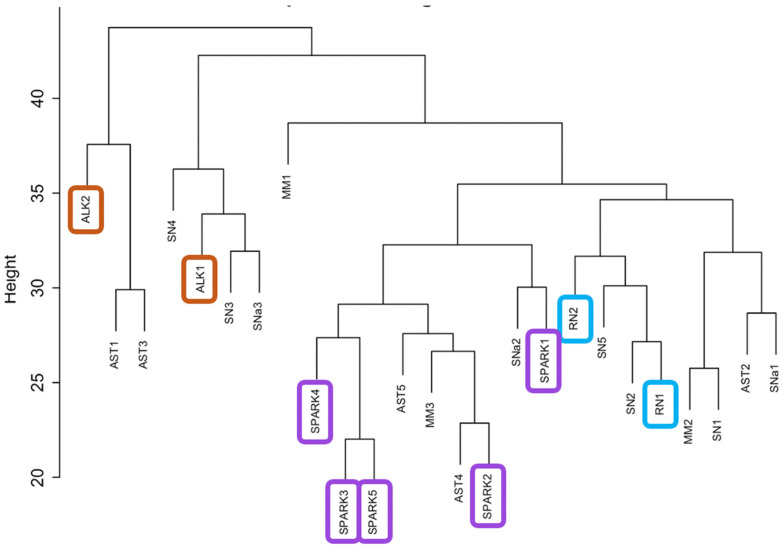
Hierarchical distance-based clustering of samples using gene expression data with the hclust function in R. Clusters for ALK-translocated tumors, SPARK nevi, and Reed nevi are highlighted in red, purple, and blue, respectively.

Gene expression analysis. We then analyzed the gene expression profile from the RNA-seq data. As shown in Figure 2, sample clustering analysis based on gene expression clearly identified the two groups of lesions driven by known gene fusions, ALK (*orange outline*) and NTRK (*blue outline*), in distinct clusters. In the presence of a known driver, ALK and NTRK fusion in these two clusters raises questions about the potential presence of a “signature” driven by these or other genes in the neighboring samples in the same clusters. So, we decided to look at the expression of ALK across all samples (Supplementary Figure S1, panel A). This analysis revealed an elevated expression in the ALK-fused samples (ALK 1 and ALK2), as expected; there was also an elevation of ALK expression in other samples (SN2, SN3, and SN5), of which only one (SN3) was clustering with the ALK translocated tumors. This finding suggests that ALK expression alone may not be sufficient for clustering those cases. We also confirmed ALK protein expression in the same lesions; the results (shown in Supplementary Figure S1, panel B) confirm expression of ALK in ALK1, SN2, SN3, and SN5.

Similarly, we looked at the expression of NTRK genes across all lesions and, as expected, the highest expression for NTRK3 was present in the Reed nevi (RN1 and RN2), although NTRK3 in the other tumors in the same cluster was not overexpressed, again implying alternative genes are involved in the reason for the clustering.

Among the other lesions, it was particularly interesting to notice that the SPARK nevi appear to cluster together (Figure 2, *purple outline*), also raising the possibility of an underlying signature in this group of lesions. Principal component analysis (PCA) (Supplementary Figure S2) showed high variability across the samples, which is typical of heterogeneous human patient samples, especially when cancerous cells are present.

Using histopathological sample classifications, we grouped samples for differential gene expression analysis but, with the limited size of the targeted capture (1385 genes), DE testing was underpowered and only previously known subtype marker genes such as ALK and TERT were found to be significantly differentially expressed (FDR < 0.05) in these comparisons (Supplementary Figure S3, *Volcano plots*). Given the limited utility of DE testing with this data set, we next applied weighted gene correlation network analysis (WGCNA).

WGCNA analysis. WGCNA is a method to identify groups of genes with correlated expression in a sample cohort. This analysis is often applied to RNA-Seq data for cancer sample cohorts. WGCNA can be useful for these types of sample cohorts because this method allows the identification of clusters (modules) of highly correlated genes without requiring sample groupings, which are needed for canonical statistical tests of differential gene expression.

**Figure 3:**
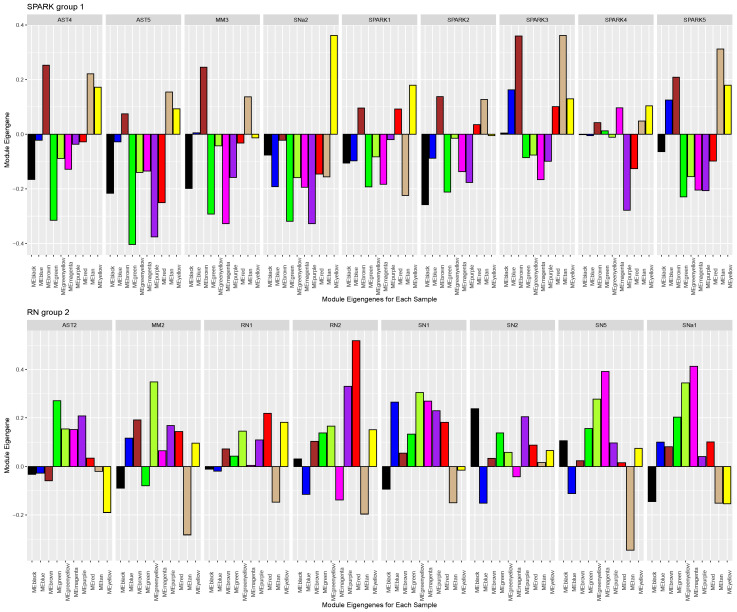

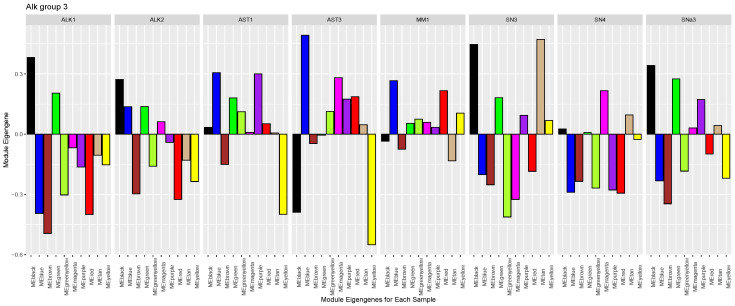
WGCNA module eigengene plots are separated by the three sample groupings: SPARK group (1), Reed nevi group (2), and ALK group (3).

Following the normalization of the gene count matrix, we applied the WGCNA dynamic module estimation and merging algorithm. Per the WGCNA convention, each module of genes is given a color name and the “grey” module consists of genes that are uncorrelated. There were 10 merged modules at the end of this analysis not including the grey module (Supplementary Table S1). We used GO enrichment analysis to identify associated pathways for the different modules (Supplementary Table S1). These modules included the brown and tan modules (enriched in transcription factors), the green–yellow module (enriched in DNA repair processes), the blue module (enriched in immune-related and plasma membrane signaling genes), the black module (enriched in growth factor receptor signaling and extracellular matrix genes), the magenta module (enriched in cell cycle regulation genes), and the yellow module (enriched in development related genes). We grouped the samples based on the initial distance-based clusters (Supplementary Figure S4) into *SPARK Group 1* (AST4, AST5, MM3, SNa1, SPARK1, SPARK2, SPARK3, SPARK4, and SPARK5), *RN Group 2* (AST2, MM2, RN1, RN2, SN1, SN2, SN5, and SNa1), and *ALK Group 3* (ALK1, ALK2, AST1, AST3, MM1, SN3, SN4, and SNa3) (Figure 3). The green–yellow (enriched in DNA repair-related genes) and the purple modules showed positive enrichment in *RN Group 2* and negative enrichment in *SPARK Group 1*. The green module showed negative enrichment in *SPARK Group 1* and positive enrichment in the other groups and the brown module (enriched in transcription factors genes) showed negative enrichment in the *ALK Group 3* cohort with positive enrichment in the other two groups.

## 4. Discussion

Histopathologic examination represents the gold standard for the interpretation and classification of the majority of melanocytic neoplasms. However, histopathology may have limitations for certain lesions, as notably exemplified by the interpretation of the Spitz family of tumors, where the histopathologic features alone are not sufficient for a unanimous and correct nosologic assessment and, more importantly, for the distinction of these lesions from malignant melanoma. To better understand these lesions, ancillary studies are sometimes necessary to better predict the prognostic trajectory and for more precise classification.

Genetic studies in the last two decades have helped to better define the category of Spitz tumors, distinctly defined by the presence of an HRAS mutation [28] or a Spitz-defining gene fusion [1,7]. Some of these driver molecular changes result in specific morphologic features and for some of these lesions, it is possible to predict the underlying genetic driver by histopathologic examination, followed by immunohistochemical validation of the specific marker (e.g., ALK). In daily practice, the use of immunohistochemistry to test for ALK, ROS1, and NTRK, as well as BRAFV600E, is a valuable screening tool, especially when there is suspicion of malignancy. Although morphologic–molecular correlation is valuable, it should be interpreted with caution because these lesions may have overlapping features and our understanding of molecular pathways in Spitz tumors is not complete. Few studies have explored gene expression profiles (GEP) of Spitz tumors [29,30,31], a methodology that may help to better understand the molecular underpinning of these lesions. Gene expression profiling has been used for other melanocytic lesions and its utility is confirmed by the use of a commercially available panel for prognostic evaluation of melanoma [32,33]. Current guidelines, like the “appropriate use criteria” released by the American Society of Dermatopathology [34], do not currently recommend the use of GEP for the interpretation of Spitz and Spitzoid lesions or other melanocytic lesions of uncertain malignant potential. However, as we expand the use of this technology on more lesions and hopefully discover useful biomarkers, there is hope for further expanding the use of GEP in the clinical setting. It has been shown that gene expression profiling can be a viable addition to evaluate difficult melanocytic lesions [35]. However, these tests need to be used with caution, as some studies, for example, have demonstrated false positivity or discordance with other types of molecular studies, specifically in the category of Spitz tumors [36]. It is also important to emphasize that no test can be used in isolation to make a definite conclusion on some of these challenging melanocytic lesions, but, rather, integration of clinical, histopathology, and ancillary studies is needed in conjunction with multi-pathologist consensus to achieve a final unifying diagnosis.

Here, we compare the morphologic classification of a spectrum of Spitz neoplasms with their RNA expression profiling and mutational profile derived from RNA sequencing data of a targeted sequencing panel including approximately 1400 cancer-related genes optimized for low-input samples like the FFPE tissues we have used in our study. This assay identified known mutations (*HRAS*) and fusions (*ALK* and *NTRK*) characteristic of Spitz tumors in a subset of our samples. *ALK* translocation-positive tumors showed increased *ALK* mRNA expression by gene expression analysis but we also noticed an increase in the expression of this gene in some non-*ALK* translocated tumors. Moreover, we confirmed this finding by immunohistochemistry. The finding of overexpression of ALK protein in Spitz lesions devoid of an ALK translocation may raise the possibility of different mechanisms of overexpression of this gene and emphasize that the use of an immunohistochemistry stain for ALK as a surrogate for ALK-translocated tumors should be interpreted with caution and only in conjunction with the characteristic histomorphology of these tumors, such as plexiform intersecting and fascicular growth pattern of fusiform melanocytes with a classical clinical presentation of a dome-shaped polypoid nodule. Similarly, our finding of overexpression of *NTRK* mRNA in Spitz tumors with no specific *NTRK* fusions may also suggest alternative mechanisms, leading to overexpression of this gene. We also attempted pan-NTRK immunohistochemical staining but we were unable to find any expression in the lesions tested. This may be due to the variable dynamic range of the pan-NTRK antibody clone at our institution, which has not been directly validated for cutaneous neoplasms. For NTRK, the analytical agreement between IHC and NGS is also complex and potentially tissue-dependent [37,38].

Our assays contained probes for other Spitz-defining translocations (including *ROS1*, *MET*, *RET*, and several *MAPK* genes) but no additional fusions were detected. Thus, these lesions may either have only Spitzoid morphology or may be true Spitz lesions with still-unknown molecular drivers.

It was particularly interesting to observe the clustering of the category of SPARK nevi based on their gene expression profile. SPARK nevi were first formally described by Ko et al. [5] as Spitz nevi with additional features of Clark or dysplastic nevi, namely cytologic atypia (of variable degree), bridging of the rete ridges and lamellar fibroplasia. Dysplastic nevi are considered intermediate lesions between benign nevi and melanoma and thus may currently be included in the category of melanocytomas. Gene expression clustering (as shown in Figure 2) grouped the SPARK nevi closely together, while terminal branches of the dendrogram included two of the ASTs. However, the remaining three ASTs were more widely distributed among our case series. Therefore, our study suggests that SPARK nevi may represent a distinct category from the AST category. SPARK nevi may have distinct underlying molecular drivers, such as the recently demonstrated *MAP2K1* mutations [39,40], as well as potentially additional molecular drivers yet to be discovered.

Our WGCNA analysis also grouped SPARK nevi together, with a similar pattern of gene modules characterizing these lesions. This finding further confirms the hierarchical distance-based clustering of samples using gene expression data. Future analysis using the entire transcriptome may help to better characterize this class of Spitz nevi, hopefully defining specific protein markers for potential immunohistochemistry analysis in a clinical setting. We also tested for modules associated with clinical variables but did not find any statistically significant associations.

A predominant limitation of this study is the sample size, which was determined by the availability of samples we could identify within a single institution. Therefore, we prioritized unbiased analyses of gene expression patterns across the entire 25-sample cohort that were not guided by the morphologic diagnosis. Another limitation was the use of a 1400-gene RNA NGS assay versus whole transcriptome sequencing. The smaller gene set of cancer-related pathways may miss additional transcriptional differences that distinguish the subtypes of Spitz and Spitzoid lesions in this cohort.

## 5. Conclusions

In conclusion, our gene expression profiling has identified distinct and known categories of Spitz and Spitzoid melanocytic lesions, further confirming the utility of using this molecular approach for the classification of these lesions. Work on larger cohorts and full transcriptome profiling will contribute to the validation of optimal GEPs or other biomarkers for clinical use. The implementation of novel predictive and prognostic biomarkers, including the use of gene expression profiles, may help in the future to better address prognostic and predictive inquiries, ultimately benefiting patients’ management.

## Figures and Tables

**Table 1:** Clinical and histopathologic characteristics of our cohort of 25 Spitz tumors. Abbreviations: M (male) and female (F).

Case ID	Consensus Diagnosis	Age (Years)	Gender	Anatomic Site	Additional Features	Follow Up	Pathogenic Fusion/Mutation
**ALK1**	Compound Spitz nevus with ALK gene fusion	19	F	Right foot	Spindled and epithelioid; mitoses	6 years (no recurrence)	TPM3-ALK fusion
**ALK2**	Compound Spitz nevus with ALK gene fusion	10	F	Right wrist	Spindled and epithelioid	(no follow up available)	TPM3-ALK fusion
**RN1**	Compound Spitz nevus/Reed Nevus	1	F	Left upper thigh	Kamino bodies; spindled	5 years (no recurrence)	MYO5A-NTRK fusion
**RN2**	Compound Spitz nevus/Reed Nevus	3	M	Left forehead	Kamino bodies; spindled	(no follow up available)	MYO5A-NTRK fusion
**SN1**	Compound Spitz nevus	2	M	Left cheeck	Kamino bodies; epithelioid (mostly) and spindled; brisk inflammation	7 years (no recurrence)	
**SN2**	Compound Spitz nevus	7	F	Left calf	Kamino bodies; spindled and epithelioid;	6 years (no recurrence)	
**SN3**	Compound Spitz nevus	3	M	Right helix	No Kamino bodies; spindled and epithelioid	5 years (no recurrence)	
**SN4**	Desmoplastic Spitz nevus (Intradermal)	19	M	R posterior neck	No Kamino bodies, 2 mitoses; spindled and epitheliod (some resemblance with ALK-fuse morphology)	5 years (no recurrence)	HRAS p.Q61K
**SN5**	Compound Spitz nevus	9	M	Right ear	Kamino bodies; spindled	4 years (no recurrence)	
**SNa1**	Compound Spitz nevus with atypia	3	M	R knee	No Kamino bodies; epithelioid and spindled; notable pagetoid array; brisk inflammtion	6 years (no recurrence)	
**SNa2**	Compound Spitz nevus with atypia	0.5 (6 months)	M	Left leg	Kamino bodies; epithelioid	(no follow up available)	
**SNa3**	Compound Spitz nevus with atypia	43	F	L sup helical rim	Spindled and epithelioid; mitoses	8 years (no recurrence)	
**SPARK1**	SPARK compound nevus	20	M	Left lower abdomen	Epithelioid and spindled	4 years (no recurrence)	
**SPARK2**	SPARK compound nevus	30	F	Right lower abdomen	Spindled and epithelioid, multifocal pagetoid scatter	5 years (no recurrence)	
**SPARK3**	SPARK compound nevus	26	F	Right thigh	Spindled and epithelioid	3 years (no recurrence)	
**SPARK4**	SPARK compound nevus	29	F	Left anterior thigh	Epithelioid, mitosis	4 years (no recurrence)	
**SPARK5**	SPARK compound nevus	31	F	Left upper mid arm	Kamino bodies; spindled and epithelioid	3 years (no recurrence)	
**AST1**	Atypical Spitz tumor	3	F	Right shoulder	No Kamino bodies; epithelioid; brisk inflammation	4 years (no recurrence)	
**AST2**	Atypical Spitz tumor	4	F	Left arm	Spindled and epithelioid; mitoses	6 years (no recurrence)	
**AST3**	Atypical Spitz tumor	18	F	Left thigh	Spindled and epithelioid	4 years (no recurrence)	HRAS p.Q61K
**AST4**	Atypical Spitz tumor	33	F	Left forearm	Epithelioid, mitosis	3 years (no recurrence)	
**AST5**	Atypical Spitz tumor	42	M	Right medial thigh	Spindled and epithelioid	2 years (no recurrence)	
**MM1**	Spitzoid Melanoma	27	M	Right mid back	Breslow 1.5 mm; 3 mitoses/mm2; no ulceration; pT2a, N2a; epithelioid; metastasis to SLN (stage IIIA)	4 years (no recurrence, no mets)	BRAF p.V600K
**MM2**	Spitzoid Melanoma	24	M	Right ear helix	Breslo 0.3 mm; no mitoses; no ulceration; pT1a; epithelioid	4 years (no recurrence)	
**MM3**	Spitzoid melanoma	53	F	Left triceps	Breslow 0.4 mm; no ulceration; pT1a; epithelioid	5 years (no recurrence)	

## Data Availability

The data presented in this study are available on request from the corresponding author.

## Supplementary Materials

The following supporting information can be downloaded at https://www.mdpi.com/article/10.3390/cancers16101798/s1. Table S1: WGCNA modules; Figure S1: (A) Gene expression of ALK, NTRK1, NTRK2, and NTRK3 in the 25 lesions. (B) Immunohistochemical analysis of ALK in ALK1, SN2, SN3, SN4, and SN5; Figure S2: PCA plot; Figure S3: Volcano plots representing gene expression comparison between groups of Spitz lesions; Figure S4: GOterm summary table for WGCNNA.
