# Supplementary figures and images for: Gene Expression Profile of Benign, Intermediate, and Malignant Spitz and Spitzoid Melanocytic Lesions
Source: Cancers (Basel). 2024 May 8;16(10):1798. doi: 10.3390/cancers16101798 (PMC11119593; doi:10.3390/cancers16101798)

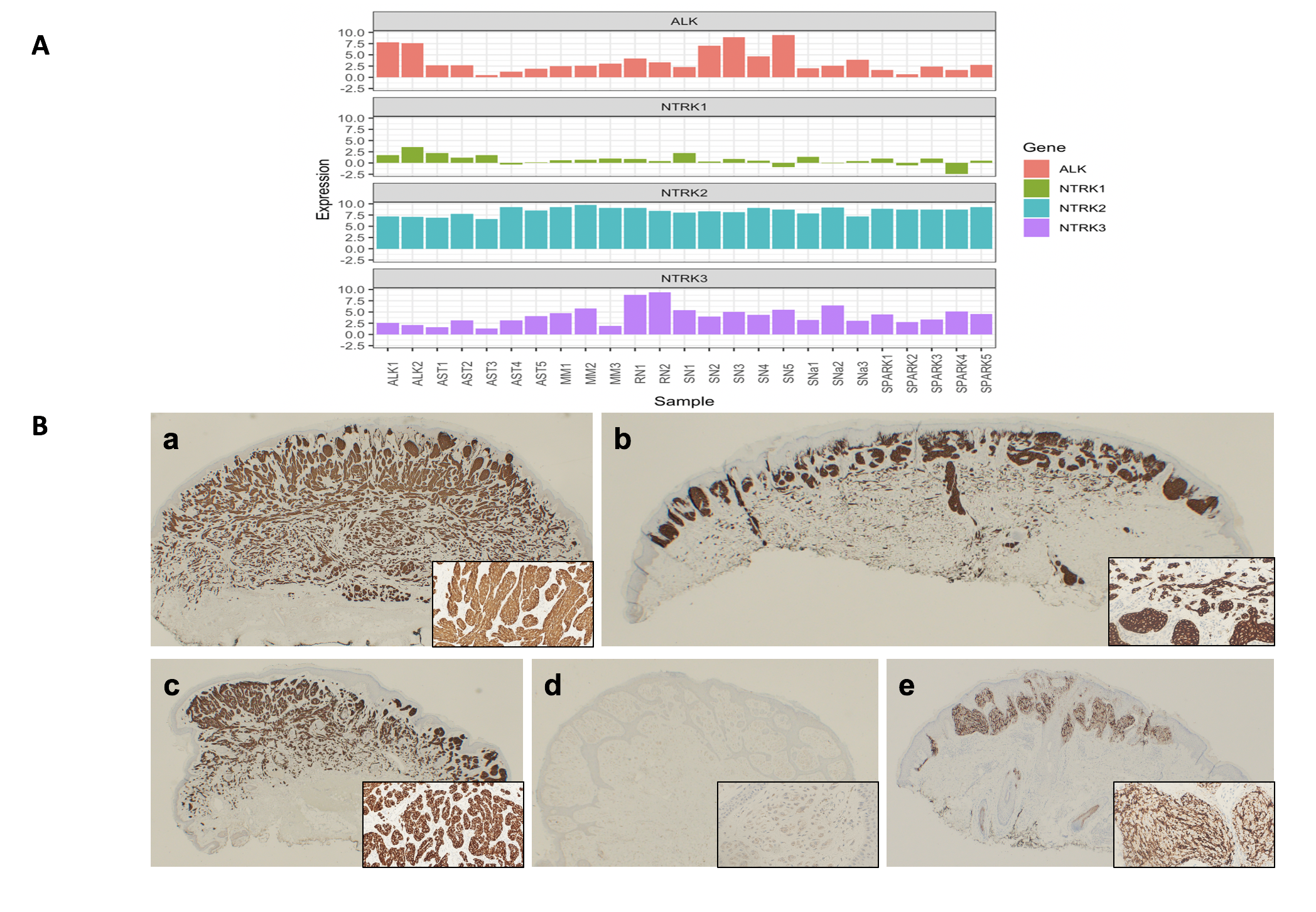

Supplement: Supplementary file 1 [file cancers-16-01798-s001.zip › New SFigure 1.png]

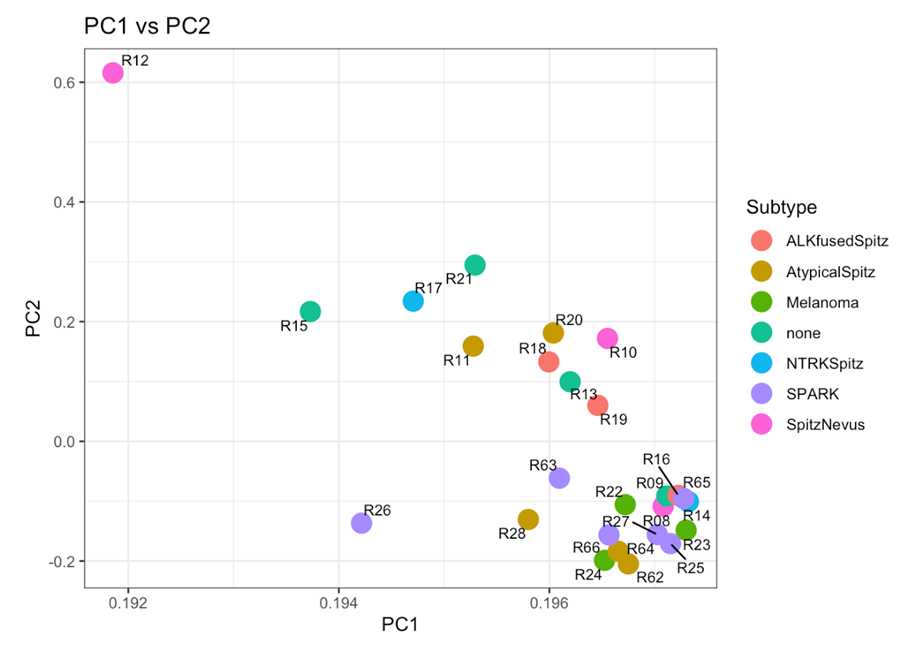

Supplement: Supplementary file 1 [file cancers-16-01798-s001.zip › SFigure 2.tif]

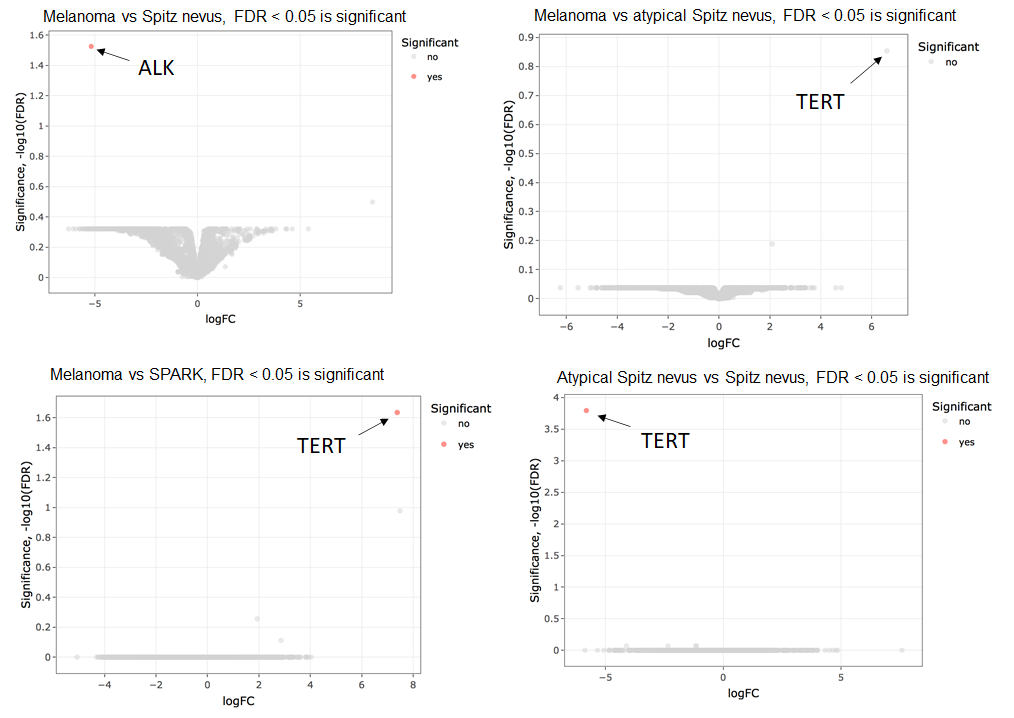

Supplement: Supplementary file 1 [file cancers-16-01798-s001.zip › SFigure 3.tif]

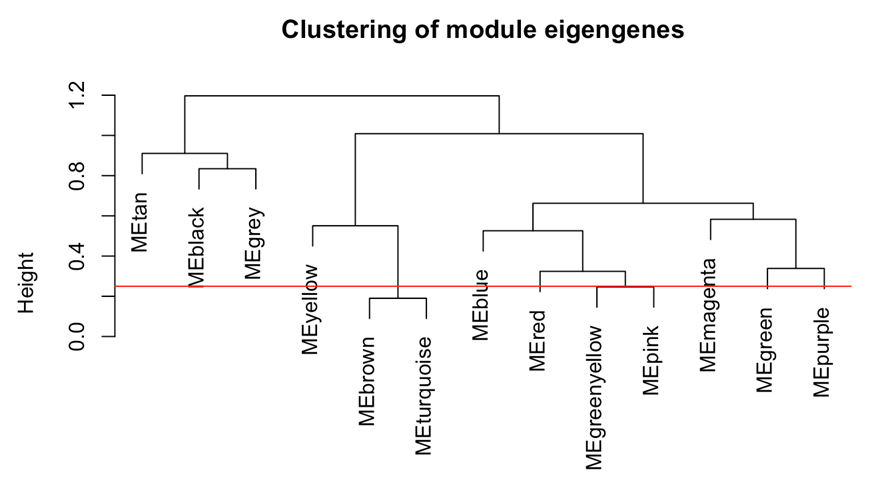

Supplement: Supplementary file 1 [file cancers-16-01798-s001.zip › SFigure 4.tif]
